# Supplementary figures and images for: How well do force fields capture the strength of salt bridges in proteins?
Source: PeerJ. 2018 Jun 11;6:e4967. doi: 10.7717/peerj.4967 (PMC6001725; doi:10.7717/peerj.4967)

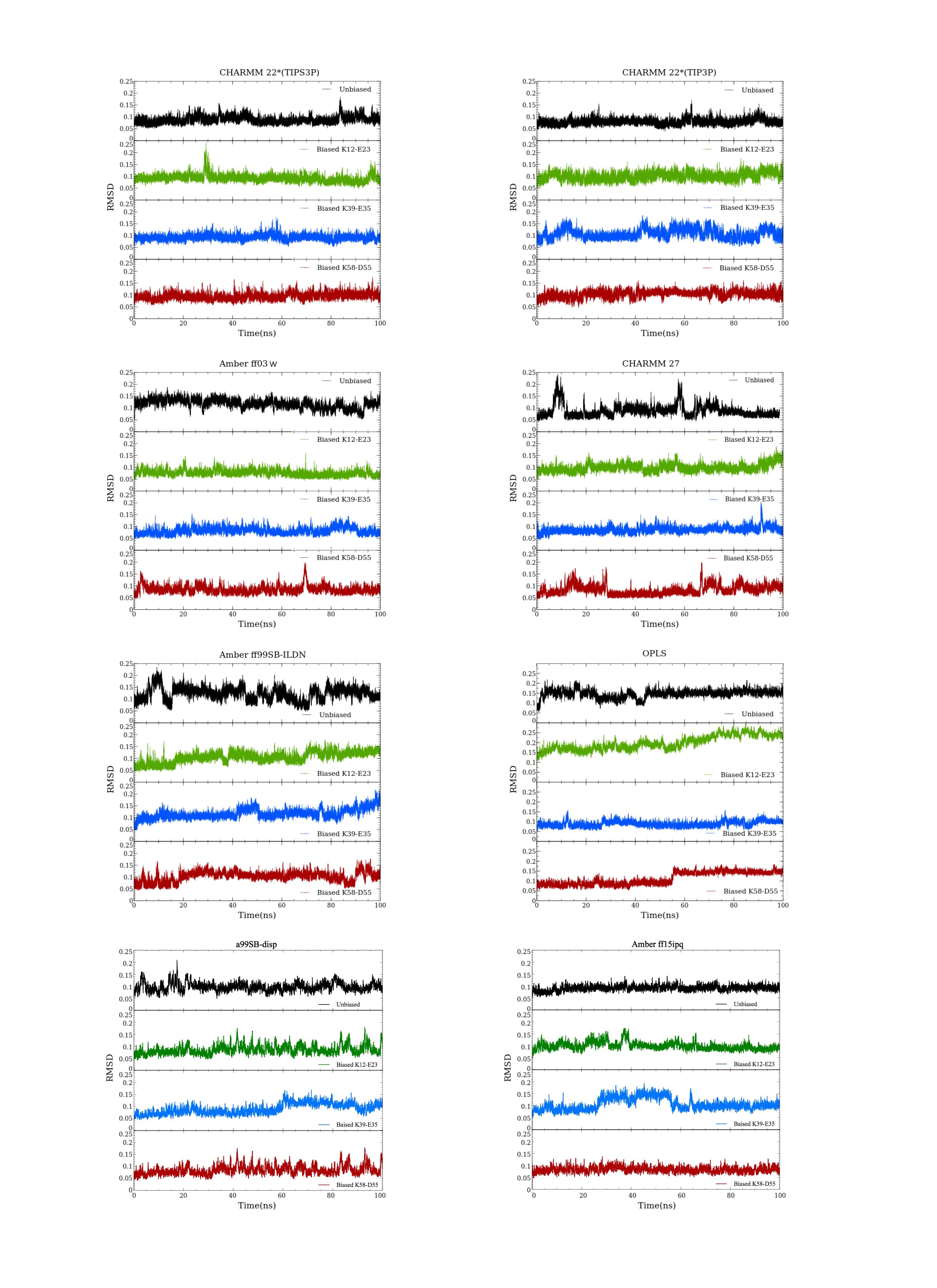

Supplement: Figure S1 — Salt bridges K12–E23 (left), K39–E35 (middle) and K58–D55 (right). [file peerj-06-4967-s001.png]

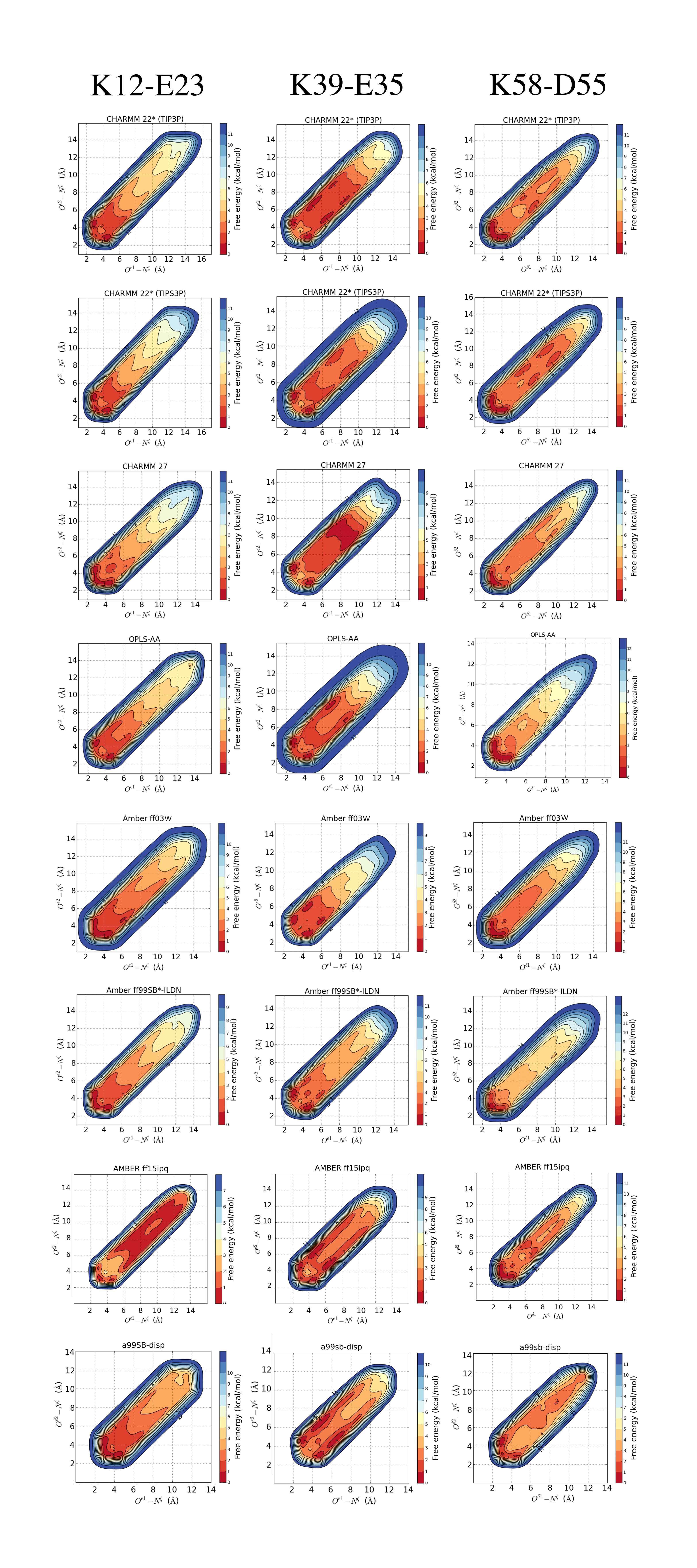

Supplement: Figure S2 — From top to bottom; unbiased, K12–E23, K39–E35 and K58–D55 biased simulation. [file peerj-06-4967-s002.png]
